# ﻿A new species of the *Baetisfuscatus* group (Ephemeroptera, Baetidae) from Morocco

**DOI:** 10.3897/zookeys.1180.109298

**Published:** 2023-09-15

**Authors:** Sara El Yaagoubi, Laurent Vuataz, Majida El Alami, Jean-Luc Gattolliat

**Affiliations:** 1 Laboratoire Ecologie, Systématique, Conservation de la Biodiversité (LESCB), Unité de Recherche Labellisée CNRST N°18, Université Abdelmalek Essaâdi, Faculté des Sciences, Département de Biologie, B.P.2121 93002 Tétouan, Morocco Université Abdelmalek Essaâdi Tetouan Morocco; 2 Muséum Cantonal des Sciences Naturelles, Palais de Rumine, Place Riponne 6, CH-1005 Lausanne, Switzerland Muséum Cantonal des Sciences Naturelles, Palais de Rumine Lausanne Switzerland; 3 University of Lausanne (UNIL), Department of Ecology and Evolution, CH-1015 Lausanne, Switzerland University of Lausanne (UNIL) Lausanne Switzerland

**Keywords:** COI, Maghreb, mayflies, morphology, Rif, taxonomy

## Abstract

*Baetisrifensis***sp. nov.** is the first representative of the *Baetisfuscatus* group to be described from the Maghreb. It was collected from streams in the Rif region of northern Morocco. All species of the *B.fuscatus* group are morphologically very similar, with slight differences in colour. Thus, in addition to morphological description, species delimitation based on genetic evidence was carried out. The new species was compared with other members of the *B.fuscatus* group from the Palaearctic region.

## ﻿Introduction

Baetidae is the most species-rich family of mayflies, with approximately 1,100 species in 114 genera (Sartori and Brittain 2015; Jacobus et al. 2019; Kaltenbach et al. 2021), representing roughly one-third of all mayfly species globally. The family is cosmopolitan, except for New Zealand (Gattolliat and Nieto 2009; Gattolliat et al. 2023). Taxonomic and hydrobiological studies are constantly adding to the list of baetid species, but knowledge remains incomplete. The mayfly fauna in Morocco comprises 54 species, with half of them belonging to Baetidae. A comprehensive checklist of these species has been recently published; however, some species are still awaiting description (El Alami et al. 2022a; Kaltenbach et al. 2022; Gattolliat et al. 2023).

**Figure 1.**
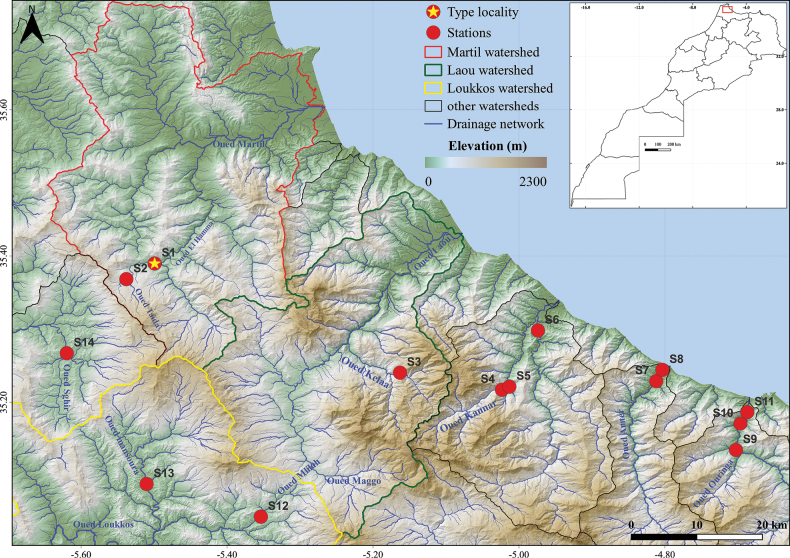
Distribution of *Baetisrifensis* sp. nov. (yellow star: type locality).

Thanks to the arduous efforts of specialists in Morocco, the country has been extensively investigated since 1990. The Rif, the solitary mountain range that emerged from the alpine orogeny and situated in the northernmost region of Morocco, has received particularly comprehensive research attention. Encompassing an area of 30,000 km^2^ (Fig. 1), the Rif stretches approximately 90 km from north to south and spans 340 km from east to west (El Alami and Dakki 1998; Errochdi et al. 2012; El Yaagoubi et al. 2023).

The genus *Baetis* Leach, 1815 presents a complicated taxonomic history, as most Baetidae with hind wings and double intercalaries in the fore wings were historically assigned to this genus. Some of the taxa were treated in the last 30 years as species groups or subgenera of *Baetis*, including taxa that are no longer considered to belong to the genus *Baetis*, such as *Labiobaetis* Novikova & Kluge, 1987 (Monaghan et al. 2005; Webb 2013; Kluge and Novikova 2016). The old concept of *Baetis* is now considered to be polyphyletic, as indicated by its division into multiple genera (e.g. Waltz and McCafferty 1987; Webb et al. 2018), which are partly based on species groups proposed for European species (Müller-Liebenau 1969). Overall, *Baetisfuscatus* and *Baetisvernus* groups are included in the genus *Baetis* s.s. according to the classification widely used in Europe (Bauernfeind and Soldán 2012; Webb et al. 2018).

The *Baetisfuscatus* group was defined by Müller-Liebenau (1969); it encompasses the European species *B.beskidensis* Sowa, 1972, *B.fuscatus* (Linnaeus, 1761), and *B.scambus* Eaton, 1870 (Bauernfeind and Soldán 2012), and the North American species *B.flavistriga* McDunnough, 1921, *B.intercalaris* McDunnough, 1921, *B.notos* Allen & Murvosh, 1987, *B.phoebus* McDunnough, 1923, and *B.rusticans* McDunnough, 1925 (Webb et al. 2018). Morphological differences between the species are very limited. The general colouration of the imagos as well as the pronotum pattern of the larvae are often used for separating the species. The *B.fuscatus* group was reported in the Eastern Palaearctic region, notably in locations such as Mongolia (Braasch 1983; Landa and Soldán 1983), Korea (Bae and Soldán 1997; Bae and Yoon 1997), China (Quan et al. 2002), and Siberia (Novikova and Kluge 1997). Unfortunately, no molecular data are currently available for these materials. Additionally, confidently attributing these populations to a specific species remains a challenging task. While a comprehensive revision of the Eastern Palaearctic species is highly recommended, it should be noted that such an endeavour falls beyond the scope of the present study.

In North Africa, the *B.fuscatus* group was first discovered in Algeria, where it was identified as *B.bioculatus*. It was later also reported from Morocco generally under *B.fuscatus* or B.gr.fuscatus (Thomas 1998; El Alami et al. 2022a). *Baetisfuscatus* group was mentioned from the Rif (El Alami 1989, 2002; El Bazi et al. 2017; Khadri et al. 2017; Guellaf et al. 2021), while in contrast, it seems to be absent in the Central Plateau and Oriental Morocco (El Agbani et al. 1992; Mabrouki et al. 2017; El Alami et al. 2022a).

In this study, we describe a new species, *Baetisrifensis* sp. nov., based on larvae collected from Rif streams. We used an integrative approach combining morphological and genetic evidence to separate the Rif populations from other Western Palearctic species.

## ﻿Material and method

By integrating all data at our disposal, we provide the distribution of *Baetisrifensis* sp. nov. in the Rif area since 1990. Specimens were collected from aquatic habitats (intermittent, ephemeral, or perennial streams) using a kick sampler during field excursions. Larvae were captured in different seasons. The data for this study came from fieldwork conducted by the first author from 2019 to 2023 and from previously collected material by El Alami between 1989 and 2018 from nearly identical sampling points.

All samples were preserved in 70% ethanol solution. The larvae were dissected and mounted on microscopic slides for morphological study under an Olympus SZX7 stereomicroscope in the Laboratoire Écologie, Systématique, et Conservation de la Biodiversité, Université Abdelmalek Essaâdi (**LESCB**).

Photographs of captured larvae were taken using a Canon EOS 6D camera and processed using Helicon Focus v. 5.3 (Helicon Soft Ltd.) and Adobe Photoshop Lightroom v. 5 (Adobe Systems Inc.). Adobe Photoshop Elements v.13 was used to enhance each image. Photographs of body parts of the larvae were taken with an Olympus BX43 microscope equipped with an Olympus SC50 camera and processed with Olympus software Cell Sense v. 1.3. The distribution map was generated using ArcGIS software (ESRI, Inc). The GPS coordinates of the sample locations are given in the sections of examined type materials.

To complement morphological investigations, we sequenced a 658-bp fragment of the mitochondrial gene cytochrome oxidase subunit 1 (COI) for five specimens of *B.rifensis* sp. nov., following the non-destructive DNA extraction procedure described in Vuataz et al. (2011). The polymerase chain reaction (PCR), purification, and sequencing steps were conducted according to the methodology described in El Alami et al. (2022b). Forward and reverse sequencing reads were assembled and edited in Codon Code Aligner v. 10.0.2 (Codon-Code Corp.) and aligned using MAFFT (Katoh et al. 2019) with default settings as implemented in Jalview v. 2.11.2.6 (Waterhouse et al. 2009). The number of parsimony-informative sites of the alignment was calculated in MegaX (Kumar et al. 2018; Stecher et al. 2020). To augment our dataset, we initially downloaded all COI sequences associated with members of the *B.fuscatus* group available on the GenBank database as of 11 May 2023, resulting in a total of 385 records. Additionally, we obtained the sequences accessible on the BOLD Systems data portal as of the same date and selectively retained only those that were not shared with GenBank, yielding an additional set of 74 sequences. We also included three additional sequences (two from Spain and one from Portugal) obtained from the unpublished European mayfly project FREDIE (https://wp.fredie.eu/). To reduce computational demand and improve gene tree readability, we then manually excluded GenBank/BOLD sequences obtained from specimens collected outside the Western Palearctic region. This selection process was conducted after confirming that the COI sequences of *B.rifensis* sp. nov. were clearly distinct from the removed sequences (data not shown). A total of 64 sequences remained for further analyses, comprising five newly generated sequences (Table 1), 55 sequences from GenBank (seven from Sroka 2012; two from Cardoni et al. 2015; two from Corse et al. 2017; 15 from Morinière et al. 2017; seven from Múrria et al. 2017; seven from Tenchini et al. 2018; 11 from Behrens-Chapuis et al. 2021; four unpublished iBOL data releases), one from BOLD (GMGMQ2692-18), and three from the project FREDIE (ES029_SR10F10; ES030_SR10G01; PT002_SR18E12).

**Table 1.** Sequenced specimens of *Baetisrifensis* sp. nov. with collection information, GenBank accessions, and nomenclature details. All specimens were nymphs from Morocco.

Specimen catalogue nb	Locality	Altitude	GPS Coordinates	Date	Collector(s)	GenBank COI	GenSeq nomenclature
GBIFCH01144168	Oued Mansoura	124 m	35.0878, -5.51028	1.IV.2021	El Yaagoubi	**OR125991**	genseq-2 COI
GBIFCH01144184	Oued El Hamma	200 m	35.3898, -5.4992	20.IV.2021	El Yaagoubi	**OR125992**	genseq-2 COI
GBIFCH01144399	Oued El Hamma	200 m	35.3898, -5.4992	20.II.2022	El Yaagoubi	**OR125995**	genseq-1 COI
GBIFCH01144398	Oued Taida	507 m	35.3684, -5.5381	27.IV.2017	El Alami	**OR125994**	genseq-2 COI
GBIFCH01144199	Oued Kelaa	400 m	35.2404, -5.1630	13.III.2021	El Yaagoubi	**OR125993**	genseq-2 COI

To explore and visualize the COI evolutionary divergence, we employed pairwise genetic distances and gene-tree approaches. COI pairwise distances were calculated using the dist.dna function from the ape 5.7-1 package (Paradis and Schliep 2019) in R v. 4.2.3 (R Core Team 2023), selecting the raw model and the pairwise.deletion option, corresponding to uncorrected *p*-distances (see Srivathsan and Meier 2012) with missing data removed in a pairwise way. Mean, minimum, and maximum distances within and between putative COI species, referred to as molecular operational taxonomic units (MOTUs) hereafter, were calculated using the ddply function from the plyr 1.8.8 package (Wickham 2011). The assignment of COI sequences to MOTUs was determined based on the results of the ASAP species-delimitation analysis (as described below). Prior to reconstructing the COI gene tree, the best evolutionary model (GTR+Γ+I) was selected based on the second-order Akaike information criterion (AICc; Hurvich and Tsai 1989) implemented in JModelTest v. 2.1.10 (Darriba et al. 2012) with five substitution schemes, six gamma categories, and default values for other parameters. To account for different substitution rates among COI codon positions, we analyzed our data set in two partitions, one with first and second codon positions, and the other with third positions (1 + 2, 3). A Bayesian-inference analysis was performed using BEAST v. 1.10.4 (Suchard et al. 2018) on the CIPRES Science Gateway 3.3 (Miller et al. 2010). The input BEAST file was generated in BEAUTi (Suchard et al. 2018), incorporating the selected evolutionary model and partition scheme described above. A relaxed molecular clock model (uncorrelated lognormal) and a UPGMA starting tree were used, with default settings for other parameters. Two independent Markov-chain Monte Carlo (MCMC) chains were run for 30 million generations, logging parameters every 1000 generations. Convergence of the MCMC runs was visually verified in Tracer v. 1.7.2 (Rambaut et al. 2018). The log and tree files from the independent runs were combined using LogCombiner v. 1.10.4 (Suchard et al. 2018), after discarding the initial 10% of trees as burn-in, ensuring that all parameters reached effective sample size values > 200. The maximum clade-credibility tree was obtained using TreeAnnotator v. 1.10.4 (Suchard et al. 2018) with default settings. Visualization and editing of the tree were conducted in iTOL v. 6.5.7 (Letunic and Bork 2021).

Finally, we applied two contrasting single-locus species delimitation methods to our COI dataset: the distance-based ASAP (Assemble Species by Automatic Partitioning; Puillandre et al. 2020) and the tree-based mPTP (multi-rate Poisson Tree Processes; Kapli et al. 2017) approaches. ASAP, an improved version of the ABGD (Automatic Barcode Gap Discovery; Puillandre et al. 2012) approach, was employed using the ASAP webserver (https://bioinfo.mnhn.fr/abi/public/asap/asapweb.html) to estimate the most probable number of MOTUs based on our COI alignment. We calculated genetic distances using simple *p*-distances and selected the species delimitation hypothesis associated to the highest barcode gap width (W) among the three partitions sharing the same best asap-score. The mPTP method, which is a multi-rate extension of the PTP (Poisson Tree Processes; Zhang et al. 2013), takes advantage of phylogenetic differences within and between species based on the number of substitutions obtained from a phylogenetic tree. We conducted mPTP using the web service available at https://mptp.h-its.org, using the BEAST COI gene trees as input (see above).

The material is deposited in the collections of the Laboratoire Écologie, Systématique, et Conservation de la Biodiversité (**LESCB**) in Tétouan, and the Muséum cantonal des sciences naturelles in Lausanne (**MZL**).

## ﻿Results

### ﻿ Baetis rifensis

Taxon classificationAnimaliaEphemeropteraBaetidae

El Yaagoubi, Vuataz & Gattolliat sp. nov.

4D649C12-E0B3-57F3-97E7-5E598849649E

https://zoobank.org/39BCB7F2-5C63-43AB-8C01-8A111337EE67

Figs 2 , 3 , 4 , 5 , 6 , 7 , 8 , 9

#### Type materials.

***Holotype*.** Morocco • 1 nymph; Tetouan Province, S1 Oued El Hamma, Loc. Jbel Laalam; 35°23'23.2"N, 5°29'57.2"W; alt. 200 m; 20.II.2022; El Yaagoubi leg.; DNA; GBIFCH01144399; MZL.

***Paratypes*.** Morocco • 38 larvae; same data as holotype; 25.III.2023; El Yaagoubi leg.; 3 on slide; 35 larvae on alcohol; LESCB • 19 nymphs; same data as holotype; 20.IV.2021; El Yaagoubi leg.; 2 on slide; LESCB and 1 larva for DNA; GBIFCH01144184; MZL • 6 nymphs; Tetouan Province, S2 Oued Taida, Loc. Beni idder; 35°22'6.10"N, 5°32'16.99"W; alt. 507 m; 28.IV.2017; El Alami leg.; DNA; GBIFCH01144398; MZL • 1 larva; Tetouan Province, S2 Oued Taida, Loc. Beni idder; 35°22'6.10"N, 5°32'16.99"W; alt. 507 m; 04.VI.2014; El Bazi leg.; LESCB • 8 nymphs; Chefchaouen Province, S3 Oued Kelaa, Loc. Akchour; 35°14'25.6"N, 5°9'46.7"W; alt. 460 m; 13.III.2021; El Yaagoubi leg.; 1 on slide; LESCB; DNA; GBIFCH01144199; MZL • 7 larvae; Chefchaouen Province, S3 Oued Kelaa, Loc. Akchour; 35°14'25.6"N, 5°9'46.7"W; alt. 460 m; 20.IX.2014; Khadri leg.; LESCB • 6 larvae; Chefchaouen Province, S4 Oued El Kannar I, Loc. Souk Lhad; 35°13'1.7"N, 5°1'24.02"W; alt. 105 m; 17.V.2014; Khadri leg.; LESCB • 4 larvae; Chefchaouen Province, S5 Oued El Kannar II, Loc. Assoul; 35°13'17.0"N, 5°0'47.2"W; alt. 68 m; 18.VI.2014; Khadri leg.; LESCB • 2 larvae; Chefchaouen Province, S6 Oued Bouhiya, Loc. Silloufene; 35°17'53.4"N, 4°58'26.3"W; alt. 35 m; 17.V.2014; Khadri leg.; LESCB • 1 larva; Chefchaouen Province, S7 Oued Amter I, Loc. Amter; 35°13'44.3"N, 4°48'42.9"W; alt. 40 m; 17.III.2014; Khadri leg.; LESCB • 5 larvae; Chefchaouen Province, S8 Oued Amter II, Loc. Amter; 35°14'39.6"N, 4°48'12.6"W; alt. 10 m; 17.III.2014; Khadri leg.; LESCB • 4 larvae; Chefchaouen Province, S9 Oued Ouringa I, Loc. Jebha; 35°8'03.4"N, 4°42'9.4"W; alt. 95 m; 17.III.2014 • 1 larva; same data as holotype; 17.V.2014; Khadri leg.; LESCB • 3 larvae; Chefchaouen Province, S10 Oued Ouringa II, Loc. Jebha; 35°10'14.2"N, 4°41'47.6"W; alt. 60 m; 17.V.2014; Khadri leg.; LESCB • 7 larvae; Chefchaouen Province, S11 Oued Ouringa III, Loc. Jebha; 35°11'11.2"N, 4°41'13.5"W; alt. 25 m; 17.III.2014; Khadri leg.; LESCB • 1 larva; same data as holotype; 17.V.2014; Khadri leg.; LESCB • 3 larvae; Chefchaouen Province, S12 Oued Mlilah, Loc. Derdara; 35°2'35.0"N, 5°21'13.0"W; alt. 198 m; 9.V.2015; El Bazi leg.; LESCB • 6 nymphs; Chefchaouen Province, S13 Oued Mansoura, Loc. Tanaqoub; 35°5'16.0"N, 5°30'37.0"W; alt. 124 m; 1.IV.2021; El Yaagoubi leg.; 1 on slide; LESCB; DNA; GBIFCH01144168; MZL • 6 larvae; Larache Province, S14 Oued Sghir, Loc. Béni Arouss; 35°16'1.63"N, 5°37'10.9"W; alt. 200 m; 02.V.2022; El Yaagoubi leg.; LESCB.

#### Description.

**Larva** (Figs 2–9). Female body length 6.0–7.8 mm; cerci 2.0–2.8 mm; median caudal filament ca 2/3 of cerci. Male body length 6.0–7.2 mm; cerci 2.2–2.8 mm; median caudal filament ca 2/3 of cerci.

**Figure 2.**
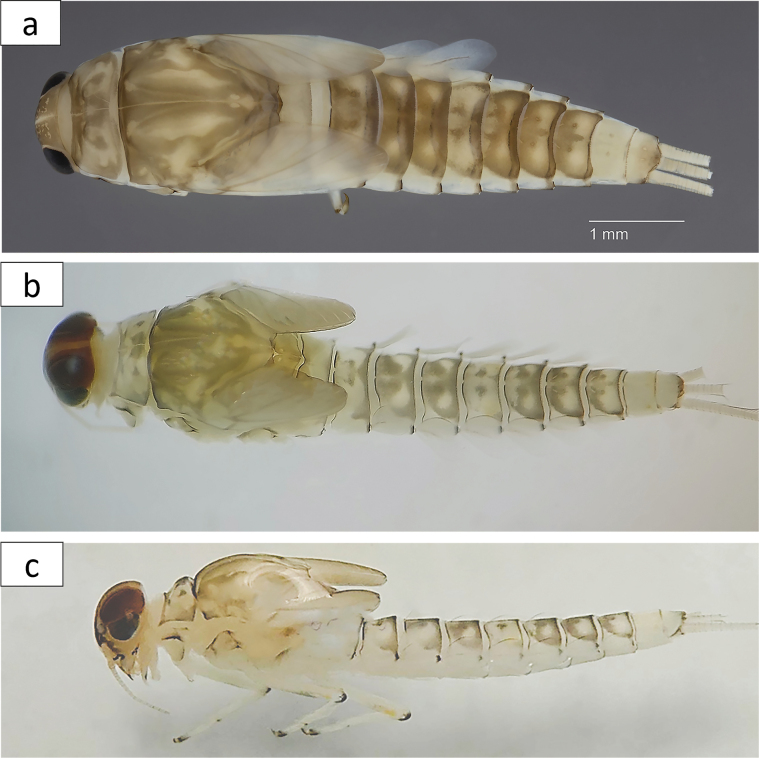
*Baetisrifensis* sp. nov., larva habitus **a** female, dorsal view **b** male, dorsal view **c** male, lateral view.

***Colouration*** (Fig. 2a–c): general colouration yellowish brown. ***Head*** uniformly brown with vermiform, yellow marking along epicranial suture, sometimes diffused and inconspicuous. ***Pronotum*** brown with dispersed swirling yellowish marks. ***Mesonotum*** brown with yellowish spots. ***Legs*** pale yellow, with a yellowish-brown patch in centre of femur and a submarginal darker stripe near outer margin of femur. ***Abdominal tergites*** medium brown and pale yellow with two central, darker brown spots: tergite I yellow; tergites II–IV brown with lateral yellow spots; tergite V yellow with lateral brown spots; tergites VI–VIII brown, yellowish distolaterally; tergites IX–X yellow; tergite X brownish apically. ***Cerci and paracercus*** yellowish or whitish yellow, with a conspicuous, dark transversal band near middle and darker apically. Basal segments sometimes also darker.

**Figure 3.**
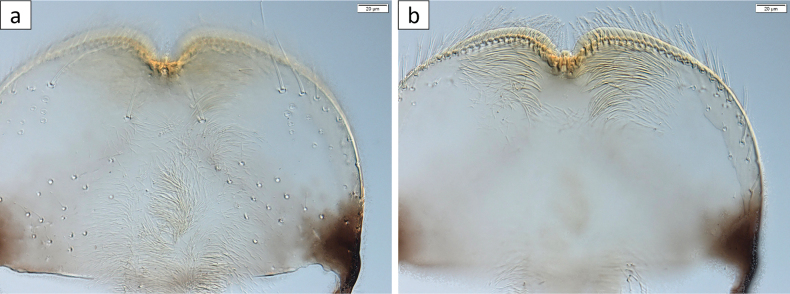
*Baetisrifensis* sp. nov., larva morphology **a** labrum dorsal face **b** labrum ventral face.

***Labrum*** (Fig. 3a, b) with 1 + (5–6) long setae, almost symmetric, evenly rounded laterally, and about 1/3 broader than long. Dorsal surface (Fig. 3a) scattered with long, pointed setae; ventral surface with apicolateral patch of long, bifid setae near margin (Fig. 3b).

**Figure 4.**
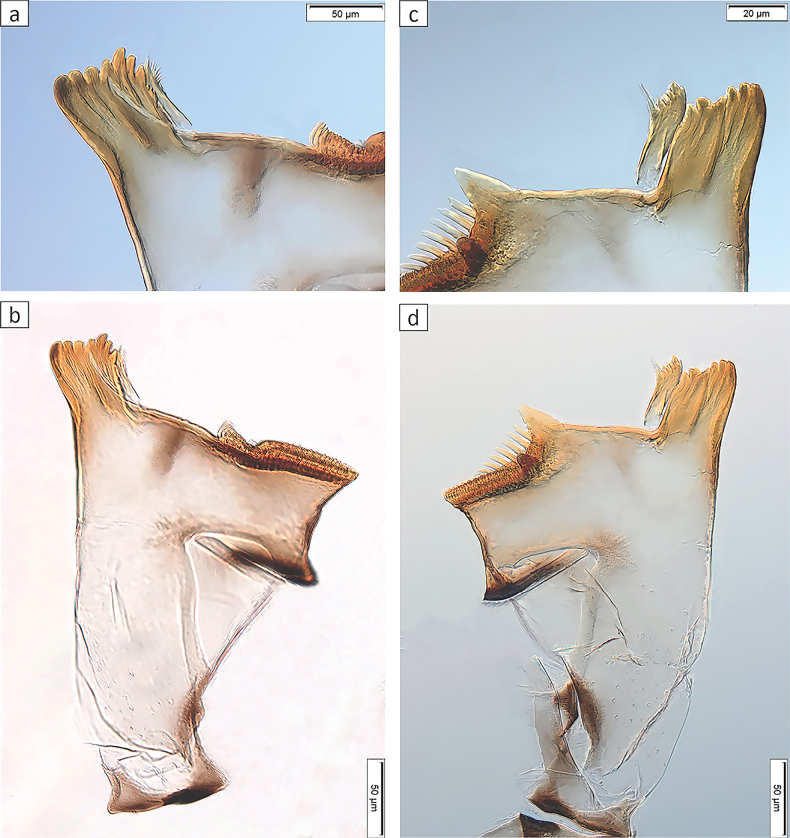
*Baetisrifensis* sp. nov., larva morphology **a, b** right mandible **c, d** left mandible.

***Right mandible*** (Fig. 4a, b): incisor and kinetodontium fused. Incisor with three apically rounded or pointed teeth subequal in size; outer tooth slightly broader. Kinetodontium with four teeth; inner margin of innermost tooth denticulate. Prostheca slender, apically pectinate.

***Left mandible*** (Fig. 4c, d): incisor and kinetodontium fused. Incisor with three apically rounded or pointed teeth subequal in size; outer tooth slightly broader. Kinetodontium with three teeth; inner margin of innermost tooth denticulate. Prostheca robust, apically with stout denticles and a comb-like structure.

**Figure 5.**
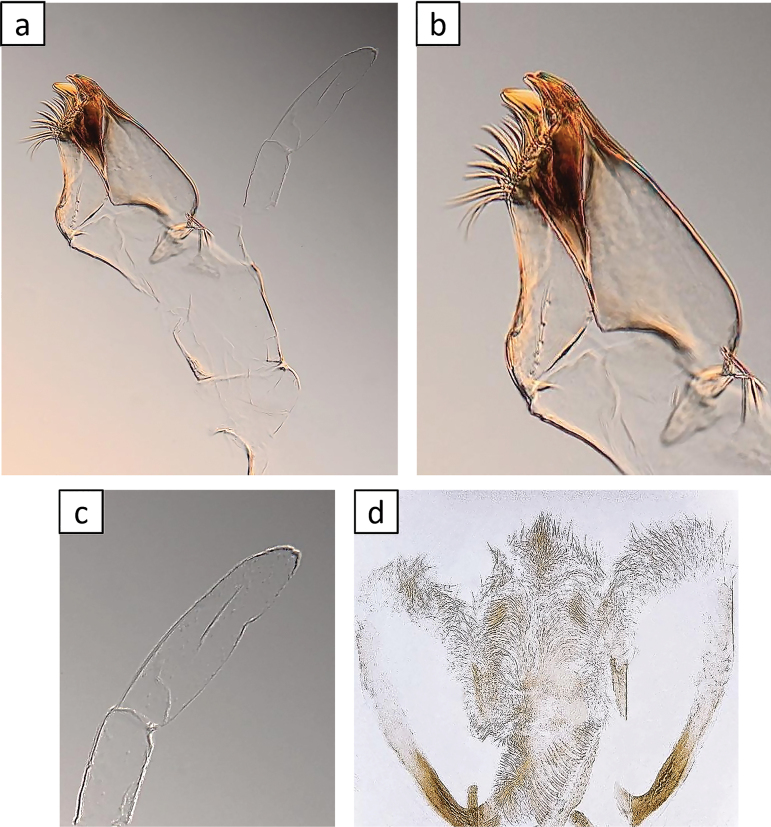
*Baetisrifensis* sp. nov., larva morphology **a** maxilla **b** apex of maxilla, **c** apex of maxillary palp, **d** hypopharynx.

***Maxilla*** (Fig. 5a, b): galea-lacinia ventrally with two simple setae under teeth. Inner dorsal row of setae with three denti-setae; distal denti-setae tooth-like; middle and proximal denti-setae slender, bifid, and pectinate.

***Maxillary palp*** (Fig. 5c) slightly longer than length of galea-lacinia; palp segment II 1.2× length of segment I; setae on maxillary palp fine, simple, scattered over surface; apex of last segment rounded.

***Hypopharynx and superlinguae*** (Fig. 5d): Lingua trilobed, apically covered with thin setae; superlingua subequal to lingua. Lingua longer than broad; medial tuft of stout setae well developed, broad; distal half laterally expanded; fine, long, simple setae along distal margin.

**Figure 6.**
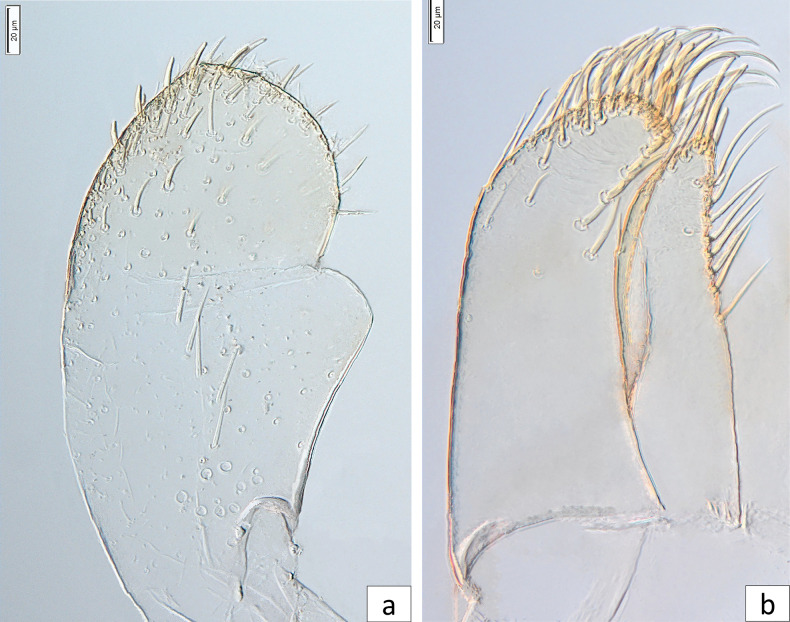
*Baetisrifensis* sp. nov., larva morphology **a** labial palp **b** paraglossae.

***Labium*** (Fig. 6a, b): glossa shorter than paraglossae; distal and lateral margins with long stout simple setae. Paraglossa (Fig. 6b) apically with three rows of long, simple, stout setae; dorsal surface with a row of five long, stout setae. Labial palps with a distinctly curved outer margin with segment I 0.7× length of segments II and III combined, segment II 1.2× length of segment I; fine, simple setae scattered over surface of segments I and II; segment II with a small, digitiform, apical projection; segment III slightly asymmetric, broadly rounded and about 1.5× broader than long; covered stout medium setae (Fig. 6a).

**Figure 7.**
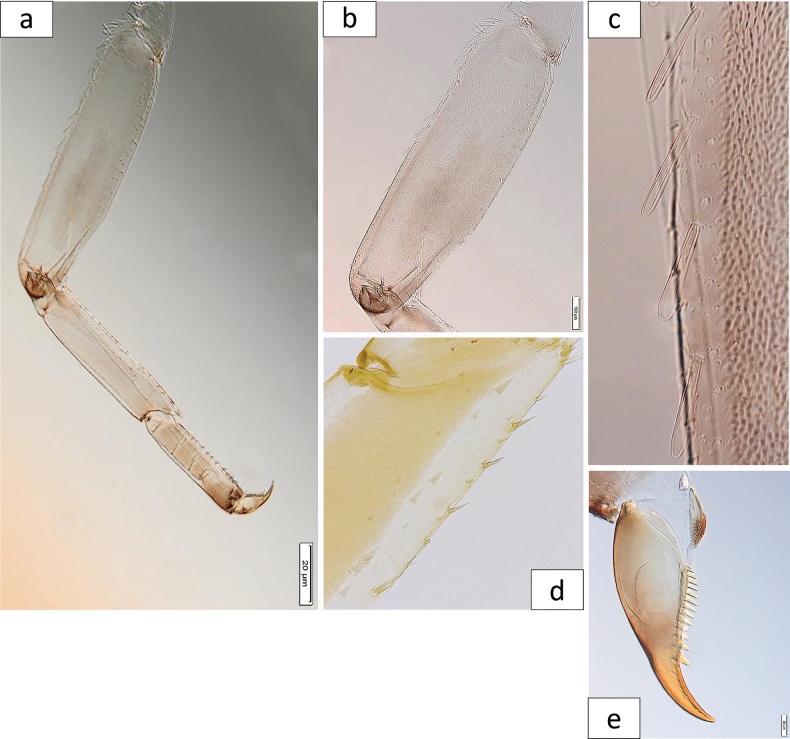
*Baetisrifensis* sp. nov., larva morphology **a** leg **b** femur **c** femur dorsal margin **d** tibia ventral margin **e** claw.

***Foreleg*** (Fig. 7a–e): ratio of foreleg segments 1.2:0.8:0.6:0.4. ***Femur*** (Fig. 7b) length 3.5× maximum width. Dorsal margin with spatulate setae (Fig. 7c), very abundant proximally, scarce distally; ventral and lateral with scattered short stout setae. ***Tibia***: dorsal margin almost bare; ventral margin with short, stout, apically pointed setae (Fig. 7d), and some fine, simple setae. ***Tarsus***: dorsal margin almost bare; ventral margin with row of curved, short to medium-length spine-like setae. ***Claw*** (Fig. 7e) with one row of 12–14 denticles, distally pointed; subapical setae absent.

**Figure 8.**
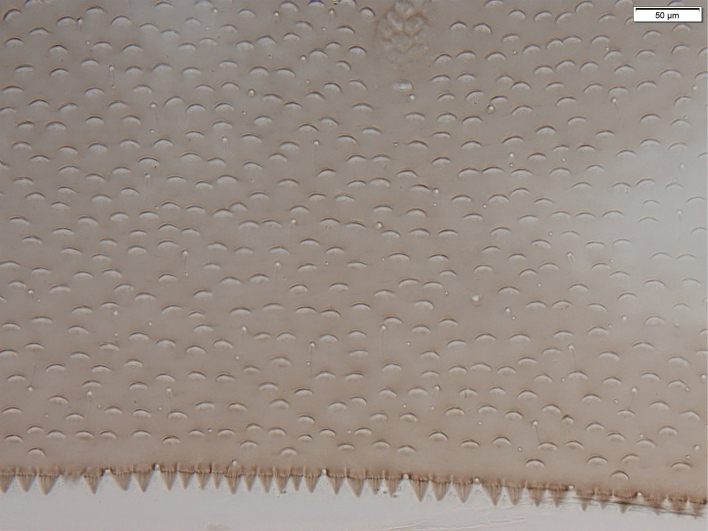
*Baetisrifensis* sp. nov., larva morphology abdominal terga V.

***Abdominal terga*** (Fig. 8): surface covered by scale bases and a few setae. Distal margins of tergites with triangular, pointed spines, longer than wide.

**Figure 9.**
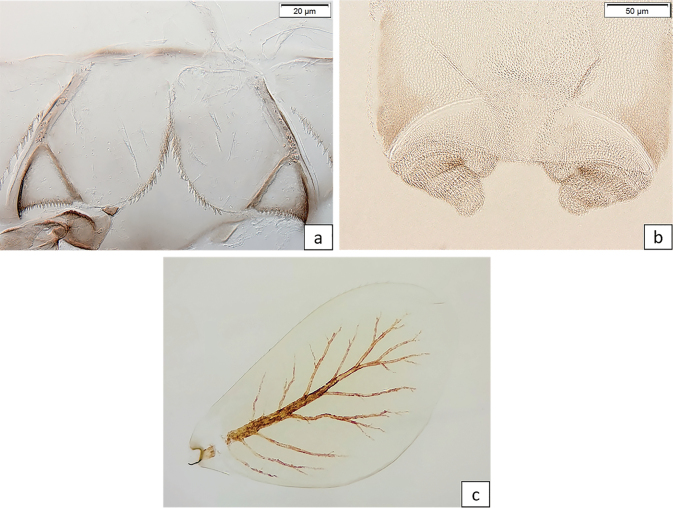
*Baetisrifensis* sp. nov., larva morphology **a** paraprocts **b** protogonostylus **c** gill.

***Paraproct*** (Fig. 9a): surface with few scale bases and broad setae; margin with ca 20 long, slender spines. Cercotractor, margin with ca 15 medium-length spines.

***Protogonostylus*** (Fig. 9b): subimaginal gonostyli developing under cuticle of last instar larvae folded in the following way: segment II bent towards middle, last segment bent laterally.

***Gills*** (Fig. 9c): tracheae extending from main trunk to inner and outer margins. Serrated costal margin without spine-like setae.

**Imagos.** Unknown.

**Subimago.** Unknown.

#### Etymology.

The new species is named after the Moroccan Rif region, from where it was collected for the first time.

#### Distribution.

Morocco: Rif (Fig. 1).

#### Biology.

The specimens were captured from rocky bottoms of moderate- to slow-velocity streams and from rhithral to potamal portions of rivers.

##### ﻿Molecular analyses

**Figure 10.**
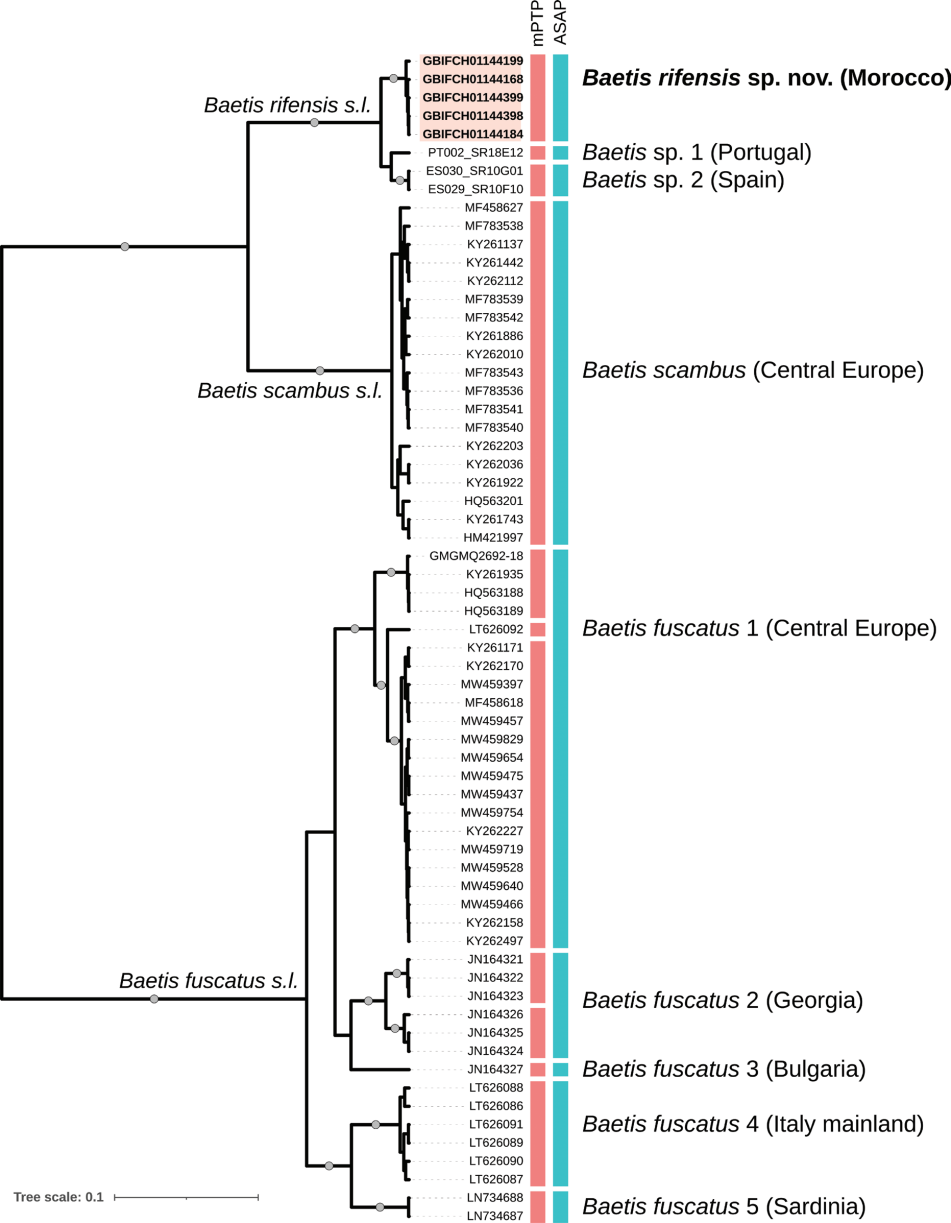
Bayesian (BEAST) maximum clade credibility COI tree of the *Baetisfuscatus* group in the Western Palaearctic. Coloured vertical boxes indicate species-delimitation hypothesis (i.e., MOTUs) according to the mPTP and ASAP methods. For each ASAP-based MOTU, the corresponding species names (where available) and the country/region of origin are provided, with the newly described species specified in bold and the associated GBIF codes highlighted in pale pink. Tips labelled with GBIF codes indicate newly sequenced specimens; PT002, ES029, and ES030 codes designate sequences from the project FREDIE; the GMGMQ2692-18 code is from BOLD; the other codes correspond to sequences obtained from GenBank. The three lineages, *Baetisrifensissensu lato* (*s.l.*), *Baetisscambuss.l.*, and *Baetisfuscatuss.l.*, are labelled above or below their respective branches. Circles on branches indicate Bayesian posterior probabilities >0.9.

The COI data set was >95% complete and included 31% of parsimony-informative sites. Pairwise COI distances across all sequences ranged from 0 to 21.8%. The overall mean *p*-distance within MOTUs was 0.8% (mean range 0–2.1%), while the overall mean *p*-distance between MOTUs was 15.4% (mean range 3.2–21.7%). The maximum *p*-distance within MOTUs varied from 0 (*Baetis* sp. 2 and *B.fuscatus* 5) to 4.3% (*B.fuscatus* 1). The minimum *p*-distance between MOTUs ranged from 3.2% (*B.rifensis* sp. nov.–*B.* sp. 1 and *B.rifensis* sp. nov.–*B.* sp. 2) to 12.2% (*B.rifensis* sp. nov.–*B.scambus*). The five sequences from *B.rifensis* sp. nov. formed a strongly supported monophyletic clade, identified as a distinct MOTU in both the ASAP and mPTP species-delimitation analyses (Fig. 10). The two species-delimitation methods were largely concordant for the other MOTUs, except for the *B.fuscatussensu lato* (*s.l.*) clade, which was split into five and eight MOTUs according to the ASAP and mPTP methods, respectively.

## ﻿Discussion

### ﻿Discriminant characters between species of the *Baetisfuscatus* group

The morphological characteristics that allow for the identification of different species within the *Baetisfuscatus* group are slight and subtle; moreover, they are often valid only for populations from a restricted area. The colouration, particularly of the pronotum and abdominal tergites, is often considered the simplest criterion. However, colouration can vary between populations and even within a population, depending on the degree of larval maturation. It is not always reliable, and the results obtained must be interpreted with caution. Although there may be variations in colour contrast between species, this pattern is generally regarded as reliable for initial identification.

The distinction between *B.fuscatus*, *B.scambus*, and *B.rifensis* sp. nov. is mainly based on the vermiform marking on the head along the epicranial suture, as well as the colouration and pattern of the pronotum and the shape of the third segment of the labial palp (Table 2). *Baetisbeskidensis* remains insufficiently known at the larval stage for reliable comparison with the new species (Müller-Liebenau 1969; Bauernfeind and Soldán 2012). However, the vermiform marks along the epicranial suture are assumed to be diffused and inconspicuous as in *B.fuscatus*, and the inner apical lobe of segment II of the labial palp is less developed than that in *B.rifensis* sp. nov. (Table 2). Moreover, the outer tooth of the right canine in *B.rifensis* sp. nov. lacks a shorter extra tooth on the outer margin that is also absent in *B.beskidensis* (fig. 1 in Sowa 1972), but it is present in *B.fuscatus* (fig. 2 in Sowa 1972; fig. 95b in Müller-Liebenau 1969).

**Table 2.** Differentiating characteristics among *Baetisrifensis* sp. nov., *B.fuscatus* and *B.scambus*.

	Baetisfuscatus*	Baetisscambus*	*Baetisrifensis* sp. nov.
Body length	5.0–6.5 mm	6.0–7.5 mm	6.0–7.2 mm
**Head**	Spots on head diffused and inconspicuous.	Spots on head along the epicranial suture conspicuous and well defined, contrastingly pale yellow or whitish.	Head uniformly brown with vermiform yellow marking along the epicranial suture, sometimes diffused and inconspicuous.
**Labial palp**	Segment 3 of labial palp slightly asymmetric, broadly rounded and about 1/3 broader than long. Inner apical lobe of segment 2 of labial palp well developed.	Segment 3 of labial palp almost symmetric, evenly rounded and about 1/3 broader than long. Inner apical lobe of segment 2 of labial palp slightly less pronounced.	Segment 3 of labial palp slightly asymmetric, broadly rounded and about 1/3 broader than long. Apical lobe of segment 2 well marked.
**Abdomen**	Abdominal terga 1, 5 and 9–10 predominantly pale, terga 2–4 and 6–8 predominantly dark with paler margin and a pair of pale diffused triangular spots (well apparent on terga 2–4).	Abdominal terga pale, dark, with paler margin and triangular spots.	Terga 1, 5 and 9–10 predominantly pale, terga 2–4 and 6–8 predominantly dark, with a narrow darker smudge near both anterior and posterior margin, and a pair of pale diffused triangular spots well apparent on terga 2–4 (often kidney shaped).

*Compiled from Müller-Liebenau (1969), Eiseler (2005), and Bauernfeind and Soldan (2012).

### ﻿Ecology and distribution

In accordance with previous investigations of Moroccan mayfly distribution, *B.fuscatus* is present across Morocco but is not particularly common (El Alami et al. 2022a). In the Rif area, the species presents a particularly broad distribution, with an elevation range of 20 to 1600 m (El Alami 2002; El Alami et al. 2022a). Currently, we can state that the species is widespread, although it appears to be less common in our recent prospects than previously found. This decline in abundance could be potentially attributed to the limited resilience of this species to recent environmental modifications. The cumulative effects of human population growth, channelization, water pollution, and water abstraction may have affected the populations. However, only further fieldwork can provide an accurate update picture of *B.rifensis* sp. nov. distribution.

**Figure 11.**
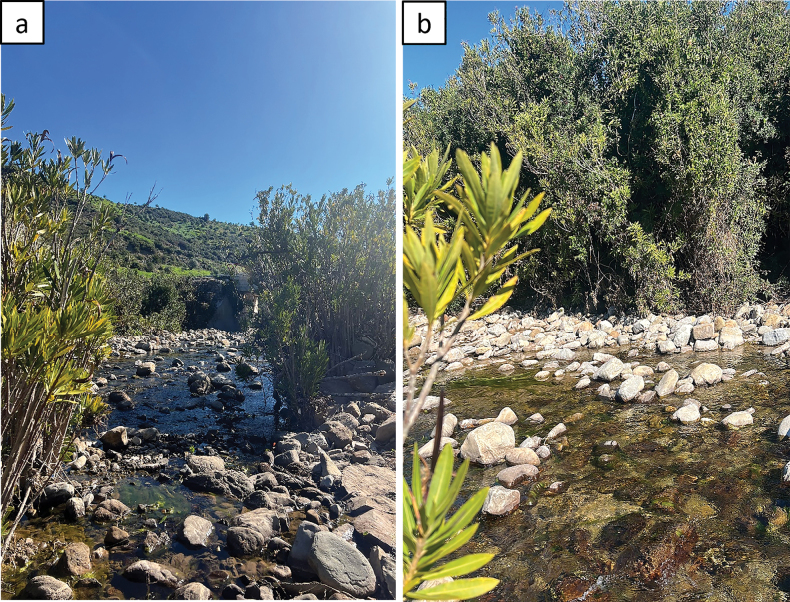
**a** global view of the type-locality: Oued El Hamma **b** habitat of *Baetisrifensis* sp. nov.

The highest density (38 larvae) was found at the type locality of Oued El Hamma on March 2023 (Fig. 11). The water temperature was 12.4 °C, dissolved oxygen 9.48 mg/l, pH 8.83, and conductivity 359 μS/cm^2^ during this time. The stream was about 5 m widt and 20 cm deep, with a moderate stream velocity of 0.6 m/s. Substrate preferences of *B.rifensis* sp. nov. seem very comparable to those of European *Baetis* species. According to earlier investigations, *B.rifensis* sp. nov. preferably occupies spring streams, upper and middle courses of wadis at middle to low altitudes, with a stony bottom rich in gravel and pebbles, which provide an excellent refuge for the larvae (El Alami et al. 2000, 2002; El Bazi et al. 2017), and with a typical affinity for temperate water between 15 and 19 °C (Khadri et al. 2017). In general, larvae of the *B.fuscatus* group are captured in rhithral to potamal sections of streams and rivers having a stony bottom and moderate- to low-velocity current (Bauernfeind and Soldán 2012).

### ﻿Genetics and biogeography

While the five sequenced specimens of *Baetisrifensis* sp. nov. form a well-supported COI clade (Fig. 10), clearly distinct from European populations of *B.scambuss.l.* and *B.fuscatuss.l.* (minimum *p*-distance of 12.2% and 19.5%, respectively), they display close phylogenetic affinity to the other members of the *Baetisrifensiss.l.* clade from Portugal (*Baetis* sp. 1) and Spain (*Baetis* sp. 2). Indeed, sequences of *B.rifensis* sp. nov. exhibit a *p*-distance to the Iberian specimens (minimum *p*-distance: 3.2%) that falls within the range typically associated with the transition from intra- to inter-species distances in mayflies (e.g. Ball et al. 2005; Kjærstad et al. 2012; Gattolliat et al. 2015). Interestingly, the distance between *Baetis* sp. 1 and *Baetis* sp. 2 (minimum *p*-distance: 3.5%) also corresponds to this transitional range. Although both the ASAP and mPTP approaches provided consistent results in dividing the members of the *Baetisrifensiss.l.* clade into three separate MOTUs, further investigations are necessary to confirm whether these MOTUs represent separate species, potentially establishing *Baetisrifensis* sp. nov. as a Moroccan endemic, or if they constitute a single species with an expanded geographical range, encompassing the Iberian Peninsula.

From a biogeographical perspective, the Betic-Rif range is one of the biodiversity hotspots of the western Mediterranean, encompassing the Iberian Peninsula and Morocco (Médail and Quézel 1997; Hajji et al. 2013). Several studies have shown that the western Mediterranean has undergone multiple events including marine transgressions and regressions during the Messinian Crisis in the late Miocene, creating hydrological corridors that may have allowed faunal exchanges between Southern Europe and the Maghreb over evolutionary time (Hsü et al. 1977; Krijgsman et al. 2002; El Alami et al. 2022a). The biological affinities between these areas attest to unique exchanges that occurred during the 260,000 years before the Messinian Salinity Crisis at the end of the Miocene, when the African and Eurasian plates were connected through the Betic-Rif corridor (Bonada et al. 2009; Blondel et al. 2010; Bemmoussat-Dekkak et al. 2021). The opening of the Strait of Gibraltar and the subsequent reflooding of the Mediterranean Sea during the Pliocene resulted from the separation of the two plates. This separation triggered the isolation and speciation of Mediterranean populations, which could explain the large number of endemic species found on either side of the Gibraltar-Mediterranean corridor (Bonada et al. 2009). Due to the complex history of the area and the short distance between continental landmasses at the Strait of Gibraltar, it is difficult to determine whether the Betic-Rifian distribution pattern is related to a common geological history or originated in recent (post-Messinian) dispersal, facilitated by the proximity and the prevailing ecological conditions in southern Iberia and North Africa (Faille et al. 2014) as reported for salamanders (Veith et al. 2004), other reptiles and amphibians (Carranza et al. 2004, 2006), and small mammals (Cosson et al. 2005). Furthermore, the Spanish-Moroccan interaction is of a great importance for understanding the biogeographical origins of many distantly related groups of Coleoptera: Scarabaeidae (Lumaret 2007) and Carabidae (Jaskuła 2015). Moreover, Trichoptera species assembly has been shaped by complex geological and paleoclimatic processes (Bonada et al. 2009; Múrria et al. 2012), promoting the predominance of Ibero-Maghrebian endemic caddisflies as a consequence of the biological association between Trichoptera in northwestern Algeria, the Rif, and Spain (Bemmoussat-Dekkak et al. 2021). Another example is Moroccan freshwater bivalves that share similar biogeographic history with Iberian clades (Froufe et al. 2016; Araujo et al. 2017; Boulaassafer et al. 2021). Overall, Casevitz-Weulersse’s (1992) and Mabrouki et al. (2020) admitted that invertebrate fauna in the Mediterranean region has stronger similarities with European Palearctic taxa than any other biogeographical region. Except for those that are endemic, all Maghreb species are found in the adjacent Mediterranean countries of Spain, Portugal, France, and Italy (Bemmoussat-Dekkak et al. 2021).

Because of the short duration of their adult stage and their restricted ability to disperse, baetids are an intriguing group for biogeographical investigations (Múrria et al. 2014). Undoubtedly, the Rif area shares more common species of mayflies with the Iberian Peninsula than any other Moroccan location; for instance, *Baetispunicus* Thomas, Boumaiza & Soldán, 1983, *Nigrobaetisrhithralis* (Soldán & Thomas, 1983), *Labiobaetisneglectus* (Navás, 1913), *Acentrellaalmohades* (Alba-Tercedor & El Alami, 1999), and *Procloeonconcinnum* (Eaton,1885) (El Alami et al. 2022a), that’s why Ibero-Maghrebian elements predominate over Moroccan endemics (El Alami 2002; Errochdi 2015; El Bazi et al. 2017; Khadri et al. 2017; Mabrouki et al. 2017; Bennas et al. 2018; Slimani 2018; Taybi et al. 2020).

## ﻿Conclusion

The widespread adoption of integrative approaches for studying Moroccan mayflies has the potential to enhance our understanding of their species diversity in the coming years. Past dubious identifications could be revised, and the description of new endemic species could be facilitated, as in the recently described species of *Prosopistomamaroccanum* (El Alami et al. 2022b) and *Centroptilumalamiae* (Kaltenbach et al. 2022). Furthermore, environmental factors go a step further in explaining the detected pattern of current mayfly distribution, as expected from ecosystems with high spatial and temporal heterogeneity, such as Mediterranean rivers. This prompts the need to address and fill the existing geographic and taxonomic gaps by directing future sampling missions to the most under-prospected locations across the entire country. This will help to remove any uncertainties in the actual identification of the genus *Baetis*.

## Supplementary Material

XML Treatment for Baetis rifensis

## ﻿Additional information

### Author ORCIDs

Sara El Yaagoubi https://orcid.org/0000-0003-1860-6433

Laurent Vuataz https://orcid.org/0000-0001-9193-8683

Majida El Alami https://orcid.org/0000-0003-2664-646X

Jean-Luc Gattolliat https://orcid.org/0000-0001-5873-5083

### Data availability

All of the data that support the findings of this study are available in the main text.
